# Repurposing 1,4-Dihydropyridine Scaffold: 4-Imidazo[2,1-*b*]thiazole-Derivatives from Calcium Entry Blockers to a New Approach for Gut Dysfunctional Motility

**DOI:** 10.3390/ph18101476

**Published:** 2025-09-30

**Authors:** Luca Camarda, Ivan Corazza, Alessandra Locatelli, Alberto Leoni, Maria Frosini, Roberta Budriesi, Emanuele Carosati, Alberto Santini, Marco Montagnani, Carla Marzetti, Laura Beatrice Mattioli

**Affiliations:** 1 Department of Pharmacy and Biotechnology (FaBiT), Food Chemistry and Nutraceutical Lab, Alma Mater Studiorum-University of Bologna, Via Belmeloro 6, 40126 Bologna, Italy; l.camarda@unibo.it (L.C.); laurabeatrice.mattioli@unibo.it (L.B.M.); 2 Department of Medical and Surgical Sciences (DIMEC), Alma Mater Studiorum-University of Bologna, 40138 Bologna, Italy; ivan.corazza@unibo.it (I.C.); marco.montagnani@unibo.it (M.M.); 3 Department of Pharmacy and Biotechnology, Alma Mater Studiorum-University of Bologna, 40126 Bologna, Italy; alessandra.locatelli@unibo.it (A.L.); alberto.leoni@unibo.it (A.L.); 4 Department of Life Sciences, University of Siena, 53100 Siena, Italy; maria.frosini@unisi.it; 5 Department of Chemical and Pharmaceutical Sciences, University of Trieste, 34127 Trieste, Italy; emanuele.carosati@units.it; 6 Valsambro S.r.l., via Cairoli 2, 40121 Bologna, Italy; alberto.santini@valsambro.it (A.S.); carla.marzetti@valsambro.it (C.M.); 7 Interdepartmental Centre for Industrial Research in Health Sciences and Technologies—CIRI Health Sciences and Technologies, Alma Mater Studiorum-University of Bologna, 40064 Bologna, Italy

**Keywords:** 1,4-dihydropyridine derivatives, drug repurposing, intestinal motility, spasmolytic activity, microbiota safety

## Abstract

**Background/Objectives**: This study investigates the pharmacological potential of 1,4-dihydropyridine derivatives, functionalized with an imidazo[2,1-*b*]thiazole scaffold, as selective modulators of intestinal motility. Given their structural similarity to both L-type calcium channel blockers and spasmolytics such as Otilonium Bromide (**OB**), we explored their repurposing for the treatment of gut motility disorders. **Methods**: A focused library of 83 1,4-dihydropyridine derivatives was screened for spasmolytic activity on potassium (80 mM)-induced depolarization in isolated guinea pig ileal and colonic tissues. Compounds showing pharmacodynamic profiles similar to **OB** and nifedipine were further evaluated for their effects on the spontaneous contractility of longitudinal and circular smooth muscle layers. Additional functional assays assessed intestinal transit, visceral nociception, and mixing/fragmentation efficiency. Microbiota safety was preliminarily tested on mixed cultures of *Bifidobacterium* and *Lactobacillus* species. **Results**: Compounds **62** and **65** selectively relaxed intestinal smooth muscle, primarily targeting the longitudinal layer without affecting vascular contractility. Ex vivo testing highlights that compounds **62** and **65** could both modulate gut transit and mixing without causing functional constipation or pain. Microbiota analyses showed no detrimental effects on “good” bacterial species *Bifidobacterium* and *Lactobacillus* spp. **Conclusions**: The favorable gastrointestinal and microbiological profiles of compounds **62** and **65**, combined with their structural versatility, support their potential repurposing for functional bowel disorders. Their selective activity suggests a promising role in therapies targeting intestinal motility while preserving microbiota homeostasis, supporting the need for extended pharmacological characterization.

## 1. Introduction

L-type calcium channels represent key therapeutic targets in numerous pathologies in which they are directly or indirectly involved. The 1,4-dihydropyridine scaffold has proven particularly effective, leading over time to the synthesis and commercialization of numerous compounds based on this scaffold, which have played a central role in the management of various cardiovascular diseases [1,2,3]. Owing to its chemical versatility, it is considered a privileged structure for potential applications In the prevention of several central nervous system (CNS) and intestinal disorders [4,5], as demonstrated by extensive research focused on both the synthesis of new derivatives and the growing understanding of the unique properties of L-type calcium channels across different tissues. Well-known derivatives such as Nifedipine (**NIF**) and Amlodipine are widely used in the treatment of hypertension.

Despite their high efficacy on L-type calcium channels in cardiovascular smooth muscle [6], these drugs are not effective for controlling intestinal motility disorders. These conditions are associated with various chronic, multifactorial gastrointestinal diseases [7] and require the use of multiple drug classes tailored to disease severity [8], combined with appropriate dietary management, as outlined in the Rome IV criteria [9]. Among these, Otilonium Bromide (**OB**) is widely used as it directly relaxes the smooth muscle of the gastrointestinal tract by blocking L-type calcium channels, thus reducing muscle contractions and relieving abdominal cramping and pain. Its local spasmolytic action, proven clinical efficacy, and favorable tolerability profile [10] make it a reference compound in the treatment of functional bowel disorders. Beyond direct L-type calcium channel blockade, intestinal motility can also be regulated through alternative receptor pathways involving calcium-dependent signaling. For example, scopolamine blocks muscarinic (at low doses) and nicotinic (at high doses) receptors in the gastrointestinal tract and ganglia, respectively, but due to poor systemic absorption, its action is mainly localized in the intestine [11]. Opioid σ receptor agonists like loperamide have limited use due to poor selectivity and the risk of inducing gallbladder atony [12]. Transient Receptor Potential Vanilloid 1 (TRPV1) channels, which are overexpressed in patients with irritable bowel syndrome (IBS), regulate transmembrane cation flow according to electrochemical gradients, causing increased intracellular Ca^2+^ and Na^+^ and subsequent depolarization [13]. Histamine H_1_-receptors located on TRPV1-expressing nerves amplify intestinal dysmotility [14], and H_1_-antagonists such as ebastine can prevent histamine-induced sensitization of TRPV1 [15]. All the above-mentioned receptors involved in the regulation of intestinal motility depend on calcium influx for activation. This highlights the potential of calcium channel blockers, which limit intracellular calcium entry, thereby reducing contractile activity and indirectly modulating the function of calcium-dependent membrane receptors [16]. Our research group has long focused on the structure–activity relationships of **NIF** analogues bearing variably substituted imidazo[2,1-*b*]thiazole moieties at position 4, aiming to develop compounds selective for specific cardiovascular parameters while considering off-target effects on intestinal smooth muscle [17,18,19,20].

Our previous work has demonstrated that the imidazo[2,1-*b*]thiazole group, variably decorated and combined with different ester substitutions at carbons 3 and 5 of the 1,4-dihydropyridine ring, can selectively modulate cardiovascular parameters, exhibit neuroprotective effects, and unexpectedly control intestinal smooth muscle activity. Notably, the 1,4-dihydropyridine scaffold substituted at position 4 with various imidazo[2,1-*b*]thiazole groups exhibits high efficacy and potency on gastrointestinal but not vascular smooth muscle [18], making it a compelling candidate for the development of new selective analogues.

**Figure 1:**
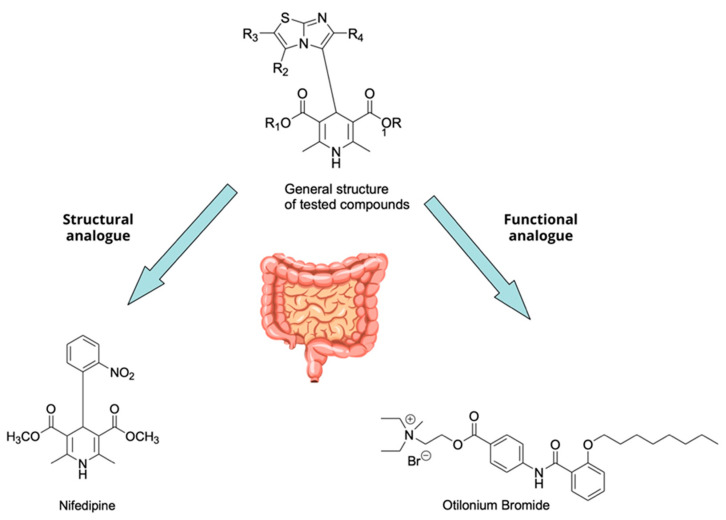
Rational of 4-Imidazo[2,1-*b*]thiazole-1,4-DHPs repurposing compounds.

In this study, we adopted a drug repurposing strategy to investigate a small chemical library of 83 compounds, originally designed for unrelated therapeutic targets, as potential modulators of intestinal motility. **NIF** was tested as a structural analogue of the synthesized compounds, given the shared 1,4-DHP core, while Otilonium Bromide (**OB**) served as a functional analogue (Figure 1). Although **NIF** is not clinically used to treat intestinal motility disorders and may even impair motility by inducing constipation [21], its inclusion allowed us to compare structural activity and selectivity profiles toward intestinal versus cardiovascular smooth muscle.

Specifically, we evaluated their effects on the contractility of longitudinal smooth muscle from rat ileum depolarized with 80 mM potassium. The most promising candidates (**2**, **10**, **31**, **43**, **62**, **65**), which did not display significant cardiovascular activity, were further characterized for their effects on potassium-depolarized smooth muscle from the colon, as well as on circular smooth muscle from both the ileum and colon.

Spontaneous intestinal contractility plays a crucial role in the digestive process, contributing to peristaltic movements that regulate the speed of bolus transit, mixing, and fragmentation [22,23]. These coordinated activities ensure that the luminal content comes into close contact with the intestinal mucosa, thereby directly influencing nutrient absorption. This function involves both the longitudinal and circular layers of the smooth muscle tissue. Given this physiological relevance, two compounds exhibiting pharmacodynamic profiles comparable to those of the reference functional analogue were selected for further investigation into spontaneous contractility.

Furthermore, their potential impact on selected populations of gut microbiota was preliminarily tested on mixed cultures of *Bifidobacterium* and *Lactobacillus* species, supporting their relevance as lead candidates for future therapeutic applications in gastrointestinal disorders.

## 2. Results

### 2.1. Chemistry

4-Imidazo[2,1-*b*]thiazole-1,4-DHPs used in this study were synthesized as previously reported. In particular, compounds **1**, **8**, **18** (**1**, **8**, **18**) [24], **2**–**7**, **9**–**10**, **12**–**16**, **19**–**20**, **22**–**24**, **26**–**39** [17], **11**, **17**, **21**, **53**–**57**, **80**–**82** [18], **25**, **59**–**79**, **83** [19], and **41**–**52**, **58** [20].

**Scheme 1:**
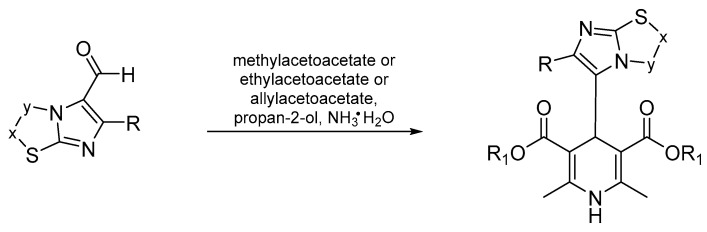
Synthesis of 4-Imidazo[2,1-*b*]thiazole-1,4-DHPs.

Briefly, the synthesis (Scheme 1) was accomplished by means of the well-known Hantzsch reaction [25]: onepot condensation of the appropriate β-ketoester, methylacetatoacetate or ethylacetoacetate or allylacetoacetate with the opportune aldehydes in a solution of aqueous ammonia and propan-2-ol.

### 2.2. Spasmolitic Activity on K^+^-Depolarized Guinea Pig Ileum Longitudinal Smooth Muscle

Table 1, Table 2 and Table 3 summarize the spasmolytic activity of the compounds on longitudinal smooth muscle from guinea pig ileum, grouped according to the esterification at positions 3 and 5 of the 1,4-dihydropyridine core. For comparison, the tables also include previously and newly reported data on cardiovascular parameters, namely negative inotropic effects on guinea pig left atrium paced at 1 Hz, negative chronotropic effects on spontaneously beating right atrium, and spasmolytic effects on K^+^-depolarized vascular smooth muscle [17,18,19,20]. For compounds not previously characterized for cardiovascular effects, intrinsic activity and potency are reported only if the intrinsic activity exceeded 50%.

Results showed that **NIF** exerted spasmolytic effects on ileal smooth muscle comparable to those observed on vascular smooth muscle. It possesses high potency on cardiovascular parameters, with a selectivity index of approximately 173-fold for vascular smooth muscle over negative inotropic activity, and about 26-fold over negative chronotropic activity. Conversely, **OB** demonstrated 230-fold lower potency on ileal longitudinal smooth muscle compared to its inotropic activity and showed no significant effects on chronotropy or vascular smooth muscle contraction.

#### 2.2.1. Methyl Esters

A total of 52 methyl esters were evaluated (Table 1). Among them, **2**, **10**, **31**, and **43** exhibited negligible activity on cardiovascular parameters while showing selective spasmolytic effects on K^+^-depolarized longitudinal ileal smooth muscle. The most potent was **10** [EC_50_ = 0.095 µM (95% CI: 0.072–0.13)], followed by **31**, which was sixfold less potent [EC_50_ = 0.55 µM (95% CI: 0.43–0.69)], then **43** [EC_50_ = 0.96 µM (95% CI: 0.74–1.06)], and **2**, which was approximately 93 times less potent [EC_50_ = 8.83 µM (95% CI: 5.53–10.41)].

All other methyl esters exhibited spasmolytic activity on ileum together with variable cardiovascular effects. Notably, only **21** displayed some activity on vascular smooth muscle and had a fivefold higher potency on ileum [EC_50_ = 0.0033 µM (95% CI: 0.0025–0.0042)] compared to vascular smooth muscle [EC_50_ = 0.016 µM (95% CI: 0.012–0.025)], and a 706-fold selectivity over inotropic effects [EC_50_ = 2.33 µM (95% CI: 1.90–2.66)].

Compounds showing negative chronotropic effects in addition to ileal spasmolytic activity included **30**, **35**, **42**, and **44**–**50**. Among these, **44**, **45**, and **50** displayed comparable potencies on both parameters. The remaining compounds were more potent on ileum than on chronotropy, with selectivity indices ranging from 8-fold (**42**) to 251-fold (**30**) and up to 461-fold (**48**). On the other hand, compounds exhibiting negative inotropic effects included **C1**, **3–8**, **15**, **17**, **23**, **26**, and **32**–**34**, and **52**. Notably, **8** showed equal potency on both parameters. Compounds **1** and **17** were selective for ileum, being 2-fold and 26-fold more potent, respectively, on intestinal smooth muscle than on inotropic activity. All other compounds in this group were more potent as negative inotropes, with varying selectivity. Compound **26** exhibited the highest selectivity for inotropic effects (approximately 281-fold).

The remaining 22 compounds (**9**, **11–14**, **16**, **18**–**20**, **22**, **24**, **25**, **27–29**, **36**–**41**, and **51**) displayed spasmolytic activity accompanied by negative inotropic and/or chronotropic effects, with variable intrinsic activity and potency. Among them, only **13**, **16**, **18**, **19**, **20**, **22**, and **24** were more potent on the ileum than on cardiac parameters. The most selective was **22**, which was 46- and 134-fold more potent on the ileum than on inotropic and chronotropic responses, respectively. Compound **16** showed the highest ileal potency [EC_50_ = 0.036 µM (95% CI: 0.028–0.046)], with 9-fold and 104-fold selectivity over inotropic and chronotropic effects, respectively. Also noteworthy was **19**, which exhibited high potency on the ileum [EC_50_ = 0.083 µM (95% CI: 0.066–0.10)] and showed 23- and 16-fold selectivity for ileal over inotropic and chronotropic parameters, respectively.

Compounds **12**, **28**, and **29** showed comparable potency across all three functional endpoints. The remaining compounds were either more potent on cardiovascular response or, as in the case of **11**, exhibited similar activity on both ileal and inotropic effects.

#### 2.2.2. Ethyl Esters

The small chemical library includes 27 ethyl ester derivatives (Table 2). Among these, only compounds **62** and **65** demonstrated selectivity for spasmolytic activity on guinea pig ileal longitudinal smooth muscle, with no significant cardiovascular effects. Both showed high intrinsic activity, exceeding 90%: 92 ± 1.4 at 10 µM for **62** and 97 ± 0.9 at 5 µM for **65**. Their potency followed a similar trend, with **65** being approximately three times more potent than **62**. As spasmolytics, both compounds were less powerful than **NIF** (by approximately 413- and 1280-fold, respectively), but more potent than **OB** (by 14- and 4-fold, respectively). All ethyl esters lacked activity on vascular smooth muscle, except for **66**, whose potency on the ileum was approximately twofold lower than its negative inotropic effect, yet four- and seventeen fold higher than its negative inotropic and chronotropic effects, respectively.

Compounds **53**, **54**, **68**, **70**, **71**, **73**, **74**, and **78** exhibited both spasmolytic activity on ileal smooth muscle and negative inotropic effects. Among them, compounds **53**, **54**, **68**, and **70** were selective for the spasmolytic effect on longitudinal ileum (by 2-, 76-, 42-, and 3-fold, respectively), while compounds **71**, **73**, **74**, and **78** were selective for negative inotropic activity (by 2-, 10-, 3-, and 10-fold, respectively). Compounds **55**, **63**, and **75** showed both spasmolytic and negative chronotropic effects. Among them, **55** was 29-fold more potent as a spasmolytic, **63** exhibited 71-fold selectivity for negative chronotropy, and **75** displayed no significant difference in potency between the two activities.

The other ethyl esters exhibited both negative inotropic and chronotropic effects. Of these, **56**–**58**, **60**, **69**, and **79** were more potent on the ileal smooth muscle than on the cardiac parameters. Notably, **79** showed a 1154-fold selectivity for the spasmolytic effect over the negative inotropic effect, but only a fourfold over negative chronotropy. Compound **67** showed comparable potency between the spasmolytic and negative inotropic effects, while **61** showed no significant difference between spasmolytic and negative chronotropic activity. Finally, the remaining, **59**, **64**, **72**, **76**, and **77**, were less potent on ileal smooth muscle than on one or both cardiac parameters.

#### 2.2.3. Allyl Esters

All the allyl ester derivatives (Table 3) tested were devoid of spasmolytic activity on vascular smooth muscle. Compounds **80**, **81**, and **83** exhibited both negative inotropic and chronotropic effects, whereas **82** showed only a negative inotropic effect. Concerning spasmolytic potency on guinea pig longitudinal ileal smooth muscle, **81** was the most potent, with an IC_50_ of 0.0053 µM (95% CI: 0.0038–0.0073), and it demonstrated marked selectivity over cardiac effects, 138-fold relative to negative inotropy and 372-fold relative to negative chronotropy. The remaining compounds were either non-selective or less potent on ileal smooth muscle than on cardiac parameters. Exceptions were **80** and **83**, which displayed higher spasmolytic potency compared to their negative chronotropic effects, by 20- and 2-fold, respectively.

#### 2.2.4. Comparative Considerations

A comparison among methyl, ethyl, and allyl esters highlights marked differences in pharmacological profiles in terms of both potency and selectivity for smooth muscle subtypes (see Table 1, Table 2 and Table 3). Methyl esters, despite a wide variability in structure, included several compounds with high spasmolytic potency on intestinal smooth muscle and limited cardiovascular involvement, suggesting a favorable selectivity profile for peripheral smooth muscle. Ethyl esters, while generally less potent than methyl analogues, featured two derivatives, **62** and **65**, that exhibited excellent selectivity for intestinal smooth muscle with negligible cardiovascular effects, underscoring their potential as gut-selective spasmolytics. Allyl esters showed the highest potencies among the series (notably **81**), yet their cardiovascular involvement was more pronounced, with fewer compounds achieving functional selectivity. Overall, these findings suggest that specific structural modifications at positions 3 and 5 of the 1,4-dihydropyridine core markedly influence the balance between intestinal and cardiovascular activity, and that selected methyl and ethyl esters may offer promising templates for the development of functionally selective smooth muscle relaxants with reduced cardiac side effects

Additionally, the comparison of compounds with the same substituent at position 4 but different esterification—methyl (**26**), ethyl (**58**), and allyl (**82**)—confirms that the combination of ester and substituent at position 4 modulates the activity profile.

We selected only those compounds that did not show significant cardiovascular effects in our experimental models. This narrow selection was necessary because, at this stage, we lack data on the bioavailability of the tested molecules. As a result, even compounds with potent and selective intestinal activity, but with concurrent cardiovascular action, were excluded from further characterization in this initial study.

### 2.3. Spasmolytic Activity on K^+^-Depolarized Guinea Pig Colon Longitudinal Smooth Muscle

The compounds with selective spasmolytic activity for the ileum longitudinal smooth muscle and without significant cardiovascular effects (**2**, **10**, **31**, **43**, **62**, and **65**), were studied on guinea pig colon longitudinal smooth muscle K^+^ depolarized (80 mM). The data are presented in Table 4, along with those of **NIF** and **OB**. **NIF** exhibited high intrinsic activity (94 ± 1.6), reaching its maximum effect at 0.01 μM, whereas **OB** achieved maximal intrinsic activity (90 ± 2.3) at 50 μM. In terms of potency, **NIF** was approximately 1805-fold more potent than **OB** [IC_50_ = 0.0019 μM (95% CI: 0.0015–0.0024) and 3.43 μM (95% CI: 2.65–4.44), respectively].

As expected, all selected compounds displayed spasmolytic activity on guinea pig longitudinal colonic smooth muscle. Among them, **65** was the most potent [IC_50_ = 0.049 μM (95% CI: 0.035–0.068)], being only 25-fold less potent than nifedipine and approximately 70-fold more potent than **OB**. **C10** showed a potency not significantly different from that of **OB**, while **2** and **31** demonstrated comparable potencies. Compounds **43** and **62** were 2-fold and 35-fold less potent than nifedipine, but 34-fold and 2-fold more potent than **OB**, respectively.

### 2.4. Spasmolitic Activity on K^+^-Depolarized Guinea Pig Ileum and Colon Circular Smooth Muscle

The selected compounds, along with the reference drugs **NIF** and **OB**, were evaluated on circular smooth muscle of guinea pig ileum and colon under identical experimental conditions. The data are summarized in Table 5. **OB** showed no significant effects on the circular muscle of either the ileum or the colon. In contrast, **NIF** was active on the circular smooth muscle of both tissues, exhibiting a 393-fold higher potency for the ileum. Only **62** and **65** displayed a profile similar to the reference drug, showing no notable activity on the circular smooth muscle of the ileum and colon. Compound **2** demonstrated selective activity on the circular smooth muscle of the ileum, with no significant effect on the colon circular muscle. Compound **43** exhibited a profile comparable to that of the structural analogue **NIF**, acting on the circular smooth muscle of both intestinal segments, with a 38-fold selectivity for the ileum. In contrast, **10** and **31** were more potent on the circular smooth muscle of the colon, with selectivity of 2.2 and 1.6 times, respectively.

### 2.5. Activity on Spontaneous Guinea Pig Ileum and Colon Smooth Muscle

Based on the spasmolytic activity data against 80 mM potassium-induced contraction, **62** and **65** were selected to investigate their effects on the spontaneous contractility of longitudinal and circular smooth muscle from guinea pig ileum and colon. Concentration–response curves were performed, and parameters such as muscle tone and peristaltic wave frequency were monitored. **NIF** and **OB** were studied in parallel under identical conditions.

#### 2.5.1. Ileum

**Figure 2:**
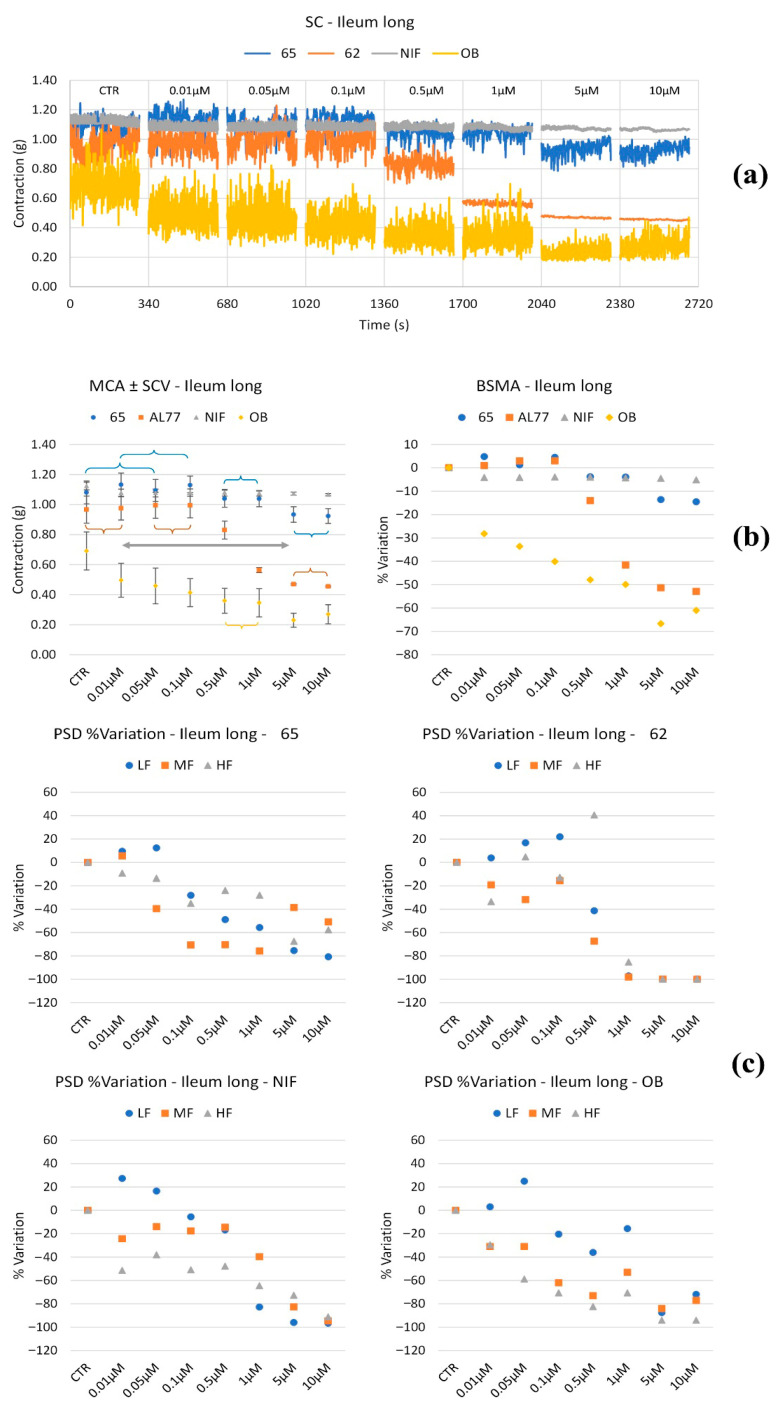
Experimental original recording of the concentration–response curve of **62**, **65**, **NIF** (Nifedipine), and **OB** (Otilonium Bromide), on spontaneous ileum longitudinal basal contractility. (**a**) Spontaneous Contraction (SC) signals for each concentration; (**b**) Mean Contraction Amplitude (MCA) and Spontaneous Contraction Variability (SCV). Not significant differences between MCAs at different concentrations are reported in the graph; all the comparisons not reported are to be considered significant (*p* < 0.05); the grey arrow highlights the not significant differences between all the tones for **NIF** for all concentrations between 0.01 μM and 10μM; (**c**) absolute powers (PSD) of the different bands of interest (LF: [0.0,0.2[ Hz; MF: [0.2,0.6[ Hz; HF: [0.6,1.0] Hz) and PSD% variations for the control phase.

*Longitudinal smooth muscle.* Figure 2 shows SC, MCA, BSMA, and PSD of spontaneous ileum longitudinal basal contractility. **NIF** showed minimal and concentration-independent effects on muscle tone. In contrast, **OB** induced a concentration-dependent reduction in longitudinal muscle tone, with a maximal decrease of approximately 70% at 5 μM. Compound **65** caused a slight tone reduction at concentrations ≥0.5 μM, while compound **62** reduced tone starting at 0.5 μM, reaching a maximum effect of approximately 50% at 5 μM. Regarding peristaltic wave frequency, **OB** decreased high-frequency (HF) and medium-frequency (MF) waves in a concentration-dependent manner; low-frequency (LF) waves were reduced by 80% at concentrations ≥5 μM. NIF, **65**, and **62** reduced LF waves starting at 1 μM, while MF and HF waves remained stable or showed only minor reductions.

**Figure 3:**
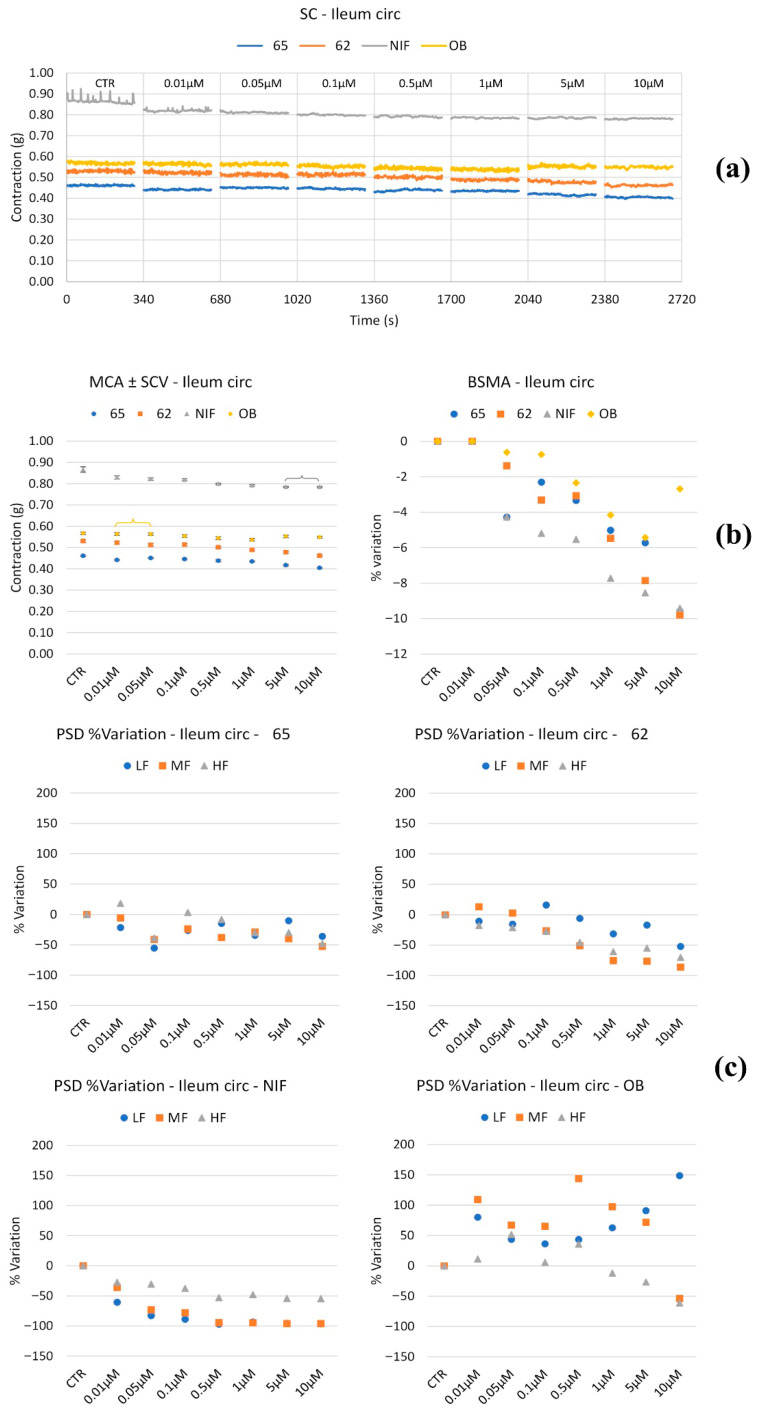
Experimental original recording of the concentration–response curve of **62**, **65**, **NIF** (Nifedipine), and **OB** (Otilonium Bromide) on spontaneous ileum circular basal contractility. (**a**) Spontaneous Contraction (SC) signals for each concentration; (**b**) Mean Contraction Amplitude (MCA) and Spontaneous Contraction Variability (SCV). Not significant differences between MCAs at different concentrations are reported in the graph; all the comparisons not reported are to be considered significant (*p* < 0.05); (**c**) absolute powers (PSD) of the different bands of interest (LF: [0.0,0.2[ Hz; MF: [0.2,0.6[ Hz; HF: [0.6,1.0] Hz) and PSD% variations with respect to the control phase.

*Circular smooth muscle*. All compounds tested (Figure 3), including the reference drugs, induced a mild reduction in muscle tone starting at 0.05 μM. Regarding peristaltic wave frequencies, **NIF**, **65**, and **62** showed minimal or no effect on low-frequency (LF), medium-frequency (MF), and high-frequency (HF) waves. In contrast, **OB** caused an increase in LF waves, peaking at 10 μM, while MF and HF waves increased at all tested concentrations but decreased by approximately 50% at 10 μM.

#### 2.5.2. Colon

**Figure 4:**
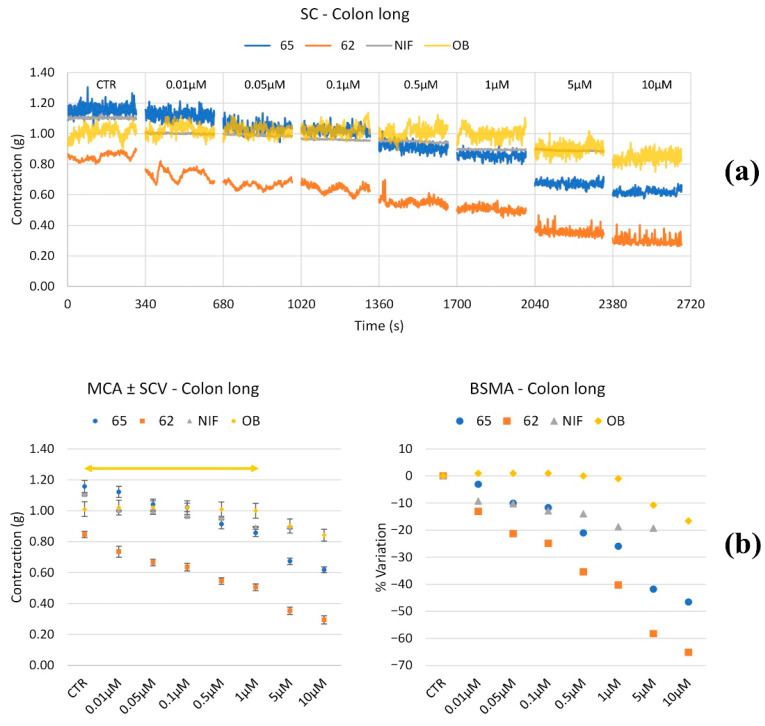

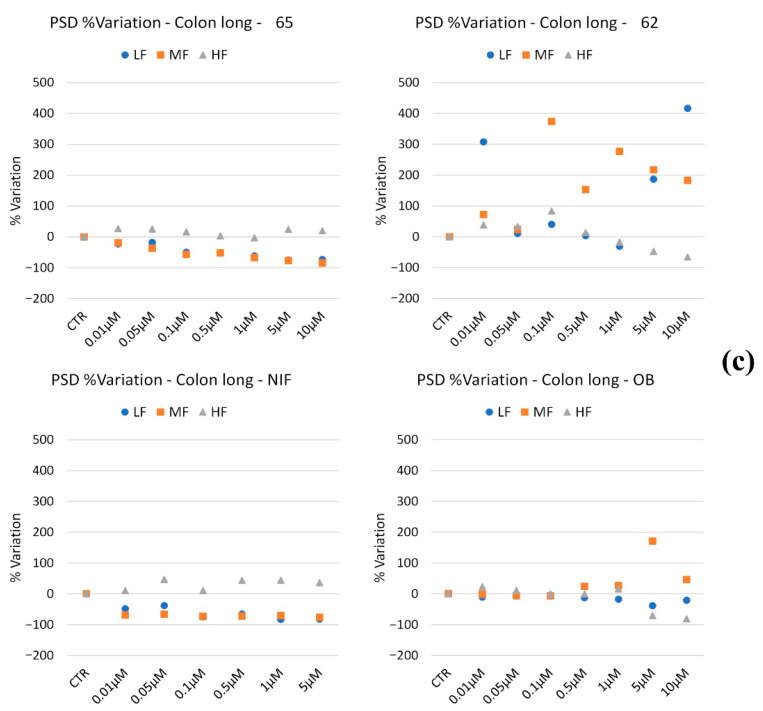
Experimental original recording of the concentration–response curve of **65**, **62**, **NIF** (Nifedipine), and **OB** (Otilonium Bromide) on spontaneous longitudinal colon basal contractility. (**a**) Spontaneous Contraction (SC) signals for each concentration; (**b**) Mean Contraction Amplitude (MCA) and Spontaneous Contraction Variability (SCV). Not significant differences between MCAs at different concentrations are reported in the graph; all the comparisons not reported are to be considered significant (*p* < 0.05); the yellow arrow highlights the not significant differences between all the tones for **OB** for all concentrations up to 1.0 μM; (**c**) absolute powers (PSD) of the different bands of interest (LF: [0.0,0.2[ Hz; MF: [0.2,0.6[ Hz; HF: [0.6,1.0] Hz) and PSD% variations with respect to the control phase.

*Longitudinal smooth muscle*. Figure 4 shows the parameters of interest of spontaneous longitudinal colon basal contractility. **NIF** caused a mild reduction in muscle tone at low concentrations, whereas **OB** reduced tone by approximately 20%, but only at concentrations above 5 μM. In contrast, compounds **62** and **65** induced a concentration-dependent tone reduction starting at low concentrations, reaching maximal decreases of –60% for **62** at 5 μM and –50% for **65** at 10 μM.

Regarding peristaltic wave frequencies, **65** mildly reduced LF and MF waves in a concentration-dependent manner, while HF waves remained stable across all concentrations. Compound **62** exhibited variable effects depending on concentration: LF waves increased by 300% at 0.1 μM, returned to control levels at intermediate concentrations, and rose again up to 400% at concentrations ≥5 μM. MF waves peaked with a 400% increase at intermediate concentrations. HF waves increased up to 0.1 μM and then progressively declined.

**NIF** decreased LF and MF waves, with HF waves remaining constant. **OB** maintained stable LF and HF waves, with decreases at high concentrations, while MF waves increased by approximately 200% only at 5 μM.

**Figure 5:**
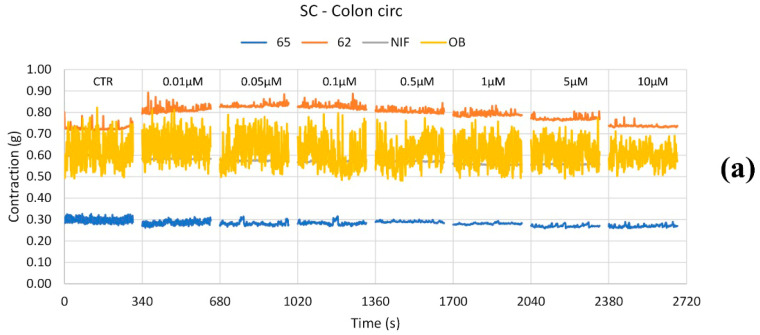

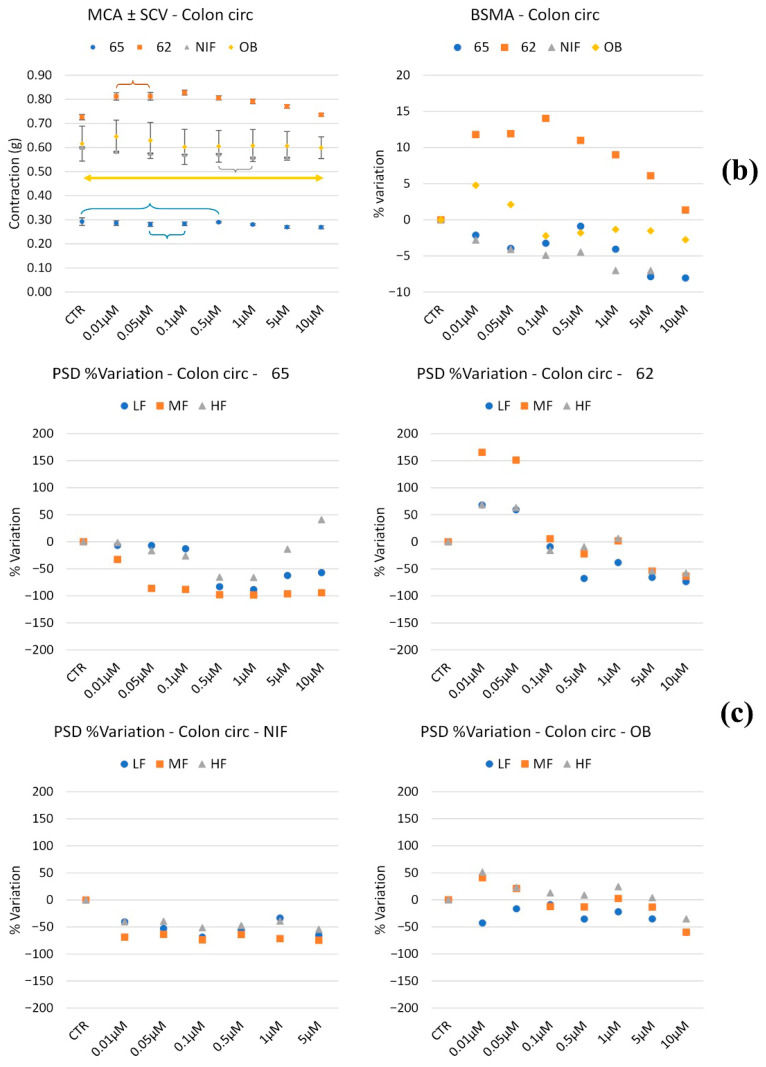
Experimental original recording of the concentration–response curve of **62**, **65**, **NIF** (Nifedipine), and **OB** (Otilonium Bromide) on spontaneous circular colon basal contractility. (**a**) Spontaneous Contraction (SC) signals for each concentration; (**b**) Mean Contraction Amplitude (MCA) and Spontaneous Contraction Variability (SCV). Not significant differences between MCAs at different concentrations are reported in the graph; all the comparisons not reported are to be considered significant (*p* < 0.05); the yellow arrow highlights the not significant differences between all the tones for **OB** for all the concentrations; (**c**) absolute powers (PSD) of the different bands of interest (LF: [0.0,0.2[ Hz; MF: [0.2,0.6[ Hz; HF: [0.6,1.0] Hz) and PSD% variations with respect to the control phase.

*Circular smooth muscle*. **OB** exhibited no significant effect on muscle tone. **NIF** and **65** caused a slight reduction in tone across all tested concentrations. Conversely, **62** induced a mild increase in tone, peaking at +15% at 0.1 μM (Figure 5).

Regarding peristaltic wave frequencies, **62** increased all frequency bands up to 0.05 μM, followed by a subsequent decrease at higher concentrations. Compound **65** reduced LF, MF, and HF waves at all concentrations, except for a slight increase in HF waves at 10 μM. **NIF** induced a mild decrease across all frequency bands, while **OB** maintained frequencies within ±50% of baseline values, showing no significant changes.

### 2.6. Effect vs. Mixed Cultures of Bifidobacterium and Lactobacillus Species

**Figure 6:**
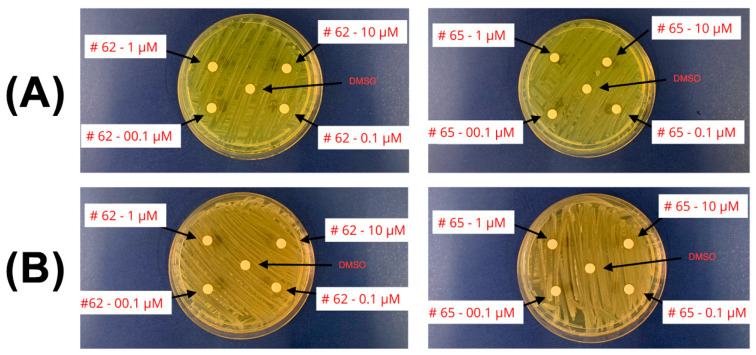
Agar plates showing no growth inhibition. (**A**) *Bifidobacteria.* (**B**) *Lactobacilli*. Concentrations of **62** and **65** are detailed.

Microbiota safety was preliminarily tested on cultures of beneficial gut bacteria to ensure that the compounds do not disrupt microbial communities essential for intestinal health and overall homeostasis. No growth inhibition was observed on any agar plate for **62** and **65** (see Figure 6), showing that **62** and **65** do not affect the viability of beneficial gut bacteria at the concentrations tested.

## 3. Discussion

Our research group has extensively investigated 1,4-dihydropyridines (DHPs) modified at position 4 by replacing the ortho-nitrobenzene moiety with diversely substituted imidazo[2,1-*b*]thiazole systems [17,19,20,24]. This structural modification has generated compounds with distinct pharmacological profiles, influencing cardiac parameters [17,24] and exhibiting neuroprotective properties [19,20], with potential therapeutic applications extending to cystic fibrosis [18]. These multifaceted activities arise from both the substitution pattern on the imidazo[2,1-*b*]thiazole core and the esterification at positions 2 and 6 of the 1,4-DHP scaffold. The 1,4-DHP nucleus is a versatile scaffold extensively utilized for the synthesis of compounds targeting multiple biological pathways across different therapeutic areas [27,28].

Within a library of 83 compounds, most displayed selective cardiovascular activities. However, a subset exhibited pronounced spasmolytic effects on potassium-induced contractions of the longitudinal smooth muscle of guinea pig ileum, with potency comparable to or surpassing that of **NIF**, a structural reference compound well characterized for its activity on smooth muscle tissue.

Moreover, several compounds demonstrated neuroprotective effects [19,20], suggesting a potential mechanistic link between peripheral modulation of intestinal smooth muscle and beneficial CNS effects. Given the recognized role of the CNS in gastrointestinal motility and the pathophysiology of motility disorders [29], these findings open promising avenues for future therapeutic development of 1,4-DHP derivatives.

Motivated by these observations, we hypothesized a possible repositioning of 1,4-DHPs bearing 4-substituted imidazo[2,1-*b*]thiazoles for the treatment of disorders associated with altered intestinal motility. We undertook a detailed investigation into the structure–activity relationship governing motility control, considering the Roma IV criteria that identify calcium channels as pertinent targets for motility regulation [30]. Many 1,4-DHPs exert potency on intestinal smooth muscle calcium channels comparable to or exceeding that on vascular smooth muscle channels. However, their clinical utility in this context is limited by poor selectivity and the predominant use of 1,4-DHPs as antihypertensive agents, where their pronounced tropism for non-vascular intestinal smooth muscle is considered an adverse side effect. Similar limitations apply to their use in neurological disorders, restricting the exploitation of the 1,4-DHP core’s full therapeutic potential.

This repositioning concept is supported by the fundamental role of intracellular Ca^2+^ concentration in intestinal smooth muscle contraction, regulated chiefly by calcium influx via voltage-dependent calcium channels (VDCCs), as evidenced by potassium-induced depolarization, and by receptor-mediated pathways indirectly modulating intracellular calcium to amplify phasic contractions [31].

Both pathways culminate in rapid contractions followed by plateau phases representing tonic and phasic components, respectively, with the tonic phase being highly sensitive to VDCC modulators [32].

Among the evaluated compounds, many exhibited high potency and selectivity for spasmolytic effects on ileal smooth muscle while retaining their cardiovascular activity. Remarkably, six compounds, four methyl esters (**2**, **10**, **31**, **43**) and two ethyl esters (**62**, **65**), were devoid of cardiovascular effects and selectively targeted the longitudinal smooth muscle of the ileum. Compounds **2** and **10** differ solely by their R substituent (CH_3_ vs. CF_3_), with the electron-withdrawing CF_3_ group significantly enhancing intrinsic activity and potency by approximately 93-fold. Compounds **31**, bearing a trimethoxyphenyl moiety, and **43**, featuring a para-pyridine substituent, showed comparable activity and potency profiles. The ethyl esters **62** and **65**, each containing halogen substituents, exhibited similar potency to their methyl ester counterparts. Notably, none of the allyl esters demonstrated selective spasmolytic activity on the longitudinal smooth muscle (Table 3).

While **NIF** showed high potency on both cardiovascular parameters and guinea pig longitudinal ileum muscle, **OB** exhibited more pronounced negative inotropic effects in our experimental models than effects on potassium-depolarized longitudinal ileum and colon muscles. **OB**, a quaternary ammonium compound with negligible systemic absorption, with no cardiovascular side effects, is widely used for intestinal motility disorders [33]. Included in the Roma IV criteria as a motility-modulating drug [34], **OB** served as a functional reference to evaluate the new compounds’ activity profiles.

**Figure 7:**
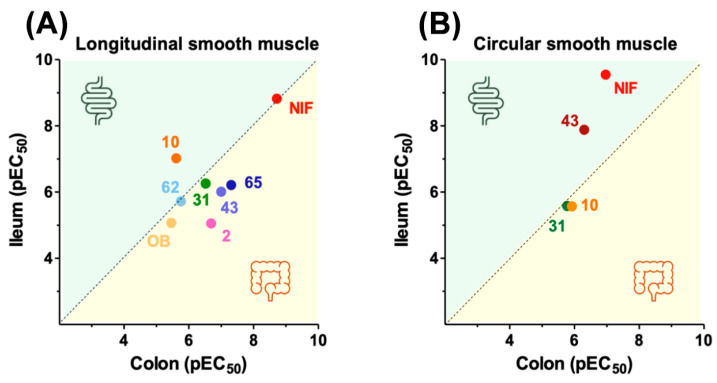
Effect of selected compounds on the ileum and colon: longitudinal smooth muscle (**A**); circular smooth muscle (**B**).

The six inactive compounds for cardiovascular parameters (**2**, **10**, **31**, **43**, **62**, and **65**) were further investigated on longitudinal colon muscle and circular ileum and colon muscles, with **OB** and **NIF** as controls. **OB** acted exclusively on induced motility of longitudinal muscle without significant effects on circular muscle, whereas **NIF** exhibited spasmolytic effects on both longitudinal and circular muscle components. As shown in Figure 7, only the two ethyl esters displayed a profile similar to **OB**. The remaining compounds affected both longitudinal and circular muscles, similar to **NIF**. Compound **43** replicated **NIF**’s selectivity, while **31** and **10** were more potent spasmolytics on circular colon muscle. **C2** selectively modulated circular ileum muscle contraction.

The characterization of compounds exhibiting a potassium depolarization activity profile similar to the functional analogue (**62** and **65**) was extended to evaluate their effects on the spontaneous contractility of longitudinal and circular smooth muscle from the ileum and colon. Table 6 and Table 7 summarize the effects produced by individual compounds at single concentrations alongside the structural reference compound (**NIF**) and the functional reference (**OB**).

In the ileum (Table 6), both **62** and **65** decrease muscle tone, inducing relaxation of both longitudinal and circular smooth muscle layers starting from a concentration of 0.5 µM. They reduce transit time at concentrations above 1 µM and may exert mild antinociceptive effects [35] while preserving mixing and fragmentation functions. These combined effects result in the modulation of intestinal transit without complete inhibition. The functional reference compound **OB** exerts a more pronounced relaxation on longitudinal muscle and mildly promotes fragmentation and mixing.

Both **62** and **65** exert relaxing effects on ileal smooth muscle, slowing bolus transit and promoting nutrient absorption, with more pronounced effects observed at concentrations ≥1 μM.

In the colon segments (Table 7), **62**, **65**, and **NIF** showed variable effects on the tone of the longitudinal smooth muscle, with **62** and **65** being more effective at lower concentrations. Both **65** and **NIF** induced relaxation of the circular muscle layer, whereas **62** elicited a contractile response. In contrast, **OB** had no significant effect on either muscle layer. Compound **65** decreased transit time without inducing nociceptive responses and reduced both mixing and fragmentation efficiency of the luminal content. Compound **62** exhibited a concentration-dependent and non-linear profile. At low concentrations, it increased transit, mixing, and fragmentation, while at intermediate concentrations, it maintained normal transit without affecting fragmentation or mixing. At high frequencies, transit was increased again, without enhancement of fragmentation or mixing at all concentrations tested; high-frequency activity was linked to discomfort or pain increase. Finally, both **NIF** and **OB** preserved normal transit velocity and nociceptive thresholds, showing only mild effects on mixing and fragmentation dynamics.

Compound **62** exerts its effects primarily on the relaxation of longitudinal smooth muscle, with no significant impact or slightly negative effects on other parameters. Compound **65** relaxes both the longitudinal and circular muscle layers, slightly reducing the mixing process, while simultaneously slowing transit, thereby promoting water absorption and fecal formation.

It is now well established that intestinal health and overall well-being are closely linked to the integrity of the gut microbiota [36]. Therefore, any therapeutic strategy should aim to preserve this microbial ecosystem. Among the most beneficial microorganisms, *Bifidobacterium* spp. [37] and *Lactobacillus* spp. play a key role in restoring eubiosis and supporting both intestinal [38,39] and brain health [40]. Probiotic supplementation is commonly employed to reestablish these beneficial populations. For example, in the context of ischemic stroke, inflammation can serve as a reparative process for neural tissue, but it also carries neurotoxic risk [41]. Gut microbiota composition has been shown to influence post-stroke inflammatory responses, suggesting that selective antimicrobial modulation could promote host-beneficial microbial communities.

Many natural and synthetic substances are able to modulate the microbiota, profoundly involving the gut–brain axis (GBA) [42,43].

The 1,4-dihydropyridine scaffold, when appropriately functionalized, possesses intrinsic antibacterial properties [44,45,46]. Notably, even amlodipine has been reported to restore intestinal barrier integrity and support gut microbial diversity in hypertensive mice with NAFLD [46], underscoring the importance of preserving commensal bacteria. Bifidobacteria and lactobacilli ferment dietary fibers in the colon, producing short-chain fatty acids (SCFAs), namely acetate, propionate, and butyrate. SCFAs have numerous protective effects on gut health: they enhance mucus production, protect against inflammation, and modulate neuroinflammation by inhibiting key enzymatic pathways implicated in Alzheimer’s disease [47].

The imidazo[2,1-*b*]thiazole core, a key feature in the molecules under investigation, displays selective antimicrobial properties without disrupting endogenous urovaginal microbiota [48]. Based on this background, **62** and **65** were preliminarily tested on bifidobacteria and lactobacilli spp. present in the composition of formulations currently used in clinical settings to support both intestinal and neurological well-being. The *Bifidobacterium* included equal amounts of *B. infantis*, which contributes to immune system development and fortifies the intestinal barrier [49]; *B. longum*, known for its anti-inflammatory effects [50]; *B. bifidum* [51]; *B. brevis*; and *B. lactis* [52]. On the other hand, the *Lactobacillus* blend consisted of *L. rhamnosus*, which modulates inflammation [53] and is important and connected to gut and brain homeostasis [54]; *L. fermentum*, which mitigates DSS-induced colitis via immune modulation and microbiota shifts [55]; *L. acidophilus*, shown to attenuate inflammation and restore gut barrier function in obesity models [56]; *L. plantarum*, associated with reduced anxiety, depression, and insomnia by modulating GBA activity [57]; and *L. salivarius*, which protects intestinal integrity and counters chemotherapy-induced mucositis [58].

This experimental setup was designed to assess the effect of the compounds not on isolated bacterial strains but on synergistic microbial communities relevant to host homeostasis and therapeutic applications.

## 4. Materials and Methods

### 4.1. Synthesis

The synthesis of the compounds studied is described. Please see above for details and references. All solvents and reagents were supplied by Aldrich Chemical Company Ltd. (Gillingham, UK) and were used as supplied, or were prepared according to the literature.

### 4.2. Gut In Vitro Tissues Activities

Male guinea pigs (200–400 g) obtained from Charles River (Calco, Como, Italy) were used. The animals were housed according to the ECC Council Directive regarding the protection of animals used for experimental and other scientific purposes (Directive 2010/63/EU of the European Parliament and of the Council) and the WMA Statement on Animal Use in Biomedical Research. All procedures followed the guidelines of the animal care and use committee of the University of Bologna (Bologna, Italy). The ethical committee authorization was reported and numbered as “Protocol 2DBFE.N.YEV” by the Comitato Etico Scientifico for Animal Research Protocols and approved by the Ministry of Health in December 2023. The animals were sacrificed by cervical dislocation, and the organ (ileum and proximal colon) required was set up rapidly under a suitable resting tension in 15 mL organ bath containing an appropriate physiological salt solution (PSS) consistently warmed (see below) and buffered to pH 7.4 by saturation with 95% O_2–_5% CO_2_ gas. Gut strip preparations were used to examine the spontaneously and K^+^-induced contractility of the compounds (0.001, 0.005, 0.01, 0.05, 0.1, 0.5, 1, 5, 10, 50, and 100 μM), first dissolved in DMSO and then diluted with PSS according to Mattioli et al. [59]. According to this procedure, the concentration of DMSO in the bath solution never exceeded 0.3%, a concentration which did not produce appreciable effects. During the generation of cumulative concentration–response curves, the next higher concentration of the compounds was added only after the preparation reached a steady state.

Guinea pig ileum. The terminal portion of ileum (3–4 cm near the ileo-caecal junction) was cleaned, and segments 2–3 cm long were set up under 1 g tension at 37 °C in organ baths containing Tyrode solution of the following composition (mM): NaCl, 118; KCl, 4.75; CaCl_2_, 2.54; MgSO_4_·7H_2_O, 1.20; KH_2_PO_4_·2H_2_O, 1.19; NaHCO_3_ 25; glucose 11. The two segments obtained (2–3 cm) were set up under 1 g tension in the longitudinal direction along the intestinal wall. Tissue was allowed to equilibrate for at least 30 min, during which time the bathing solution was changed every 10 min. The circular musculature was studied with a similar procedure.

Guinea pig proximal colon. Starting approximately 1 cm distal from the caecocolonic junction, two segments of about 1 cm of the guinea pig proximal colon were obtained. The proximal colon was cleaned by rinsing it with a De Jalon solution of the following composition (mM): NaCl, 155; KCl, 5.6; CaCl_2_, 0.5; NaHCO_3_, 6.0; glucose, 2.8. The mesenteric tissue was then removed. The two segments of longitudinal or circular smooth muscle were suspended in organ baths containing gassed warm de Jalon solution under a load of 1 g, maintained at 37 °C.

#### 4.2.1. Potassium-Induced Spasmolytic Activity

The experiments were carried out using a height K^+^ concentration as previously described [60]. Briefly, after the equilibration period, ileum strips (longitudinal or circular) were contracted by washing in PSS containing 80 mM KCl (equimolar substitution of K^+^ for Na^+^). When the contraction reached a plateau (about 30 min), cumulative concentration curves of compounds were constructed. Contractions were recorded using a displacement transducer (FT. 03, Grass Instruments, Quincy, MA, USA) using Power Lab software (7 Pro software; AD Instruments Pty Ltd., Castle Hill, Australia).

#### 4.2.2. Spontaneous Contraction

For the ileum and proximal colon, the tracing graphs of spontaneous contractions of longitudinal or circular ileum and colon smooth muscle were continuously recorded with LabChart Software (7 Pro software; AD Instruments, Bella Vista, New South Wales, Australia) as previously described [59,61]. After the equilibration period (approximately 30 to 45 min, depending on the tissue), cumulative concentration curves of the compounds were constructed using **NIF** and **OB** as positive controls. A 5-min stationary interval of the Spontaneous Contraction (SC) recording was selected at the final stage of each concentration. For each interval, the following parameters were extracted and calculated: the Mean Contraction Amplitude (MCA), evaluated as the mean force value (g); the standard deviations of the force values over the period, as an index of the Spontaneous Contraction Variability (SCV); the Basal Spontaneous Motor Activity (BSMA), as the percentage (%) variation of each mean force value (g) concerning the control period.

The spontaneous contractions were investigated in the frequency domain through a standard FFT analysis and a subsequent Power Spectral Density (PSD) plot. The absolute powers of the following frequency bands of interest, low [0.0,0.2[ Hz (LF), medium [0.2,0.6[ Hz (MF), and high [0.6,1.0] Hz (HF) [59], were then calculated. The PSD percentage (%) variations for each band of interest concerning the control were estimated.

All calculations were performed during the post-processing phase. To avoid errors due to the presence of artifacts, the period of analysis was chosen by a skilled operator.

#### 4.2.3. Cardiovascular Activity

Heart. The methods related to the study of inotropic and chronotropic effects evaluated separately on guinea pig left atria driven at 1 Hz and on spontaneously beating right atria, respectively, have been previously described [62].

Aorta. As previously described [62], the thoracic aorta was prepared and used precontracted with 80 mM K^+^ (see above) to evaluate its spasmolytic activity.

#### 4.2.4. Data Analysis

For the spasmolytic activity on K^+^-depolarized gut strips, data were analyzed using Student’s *t*-test and are presented as mean ± S.E.M. [26]. Since the drugs were added cumulatively, the difference between the control and the experimental values at each concentration was tested for a *p* value < 0.05. The potency of drugs, defined as IC_50_, was evaluated from log concentration–response curves (Probit analysis using Litchfield and Wilcoxon [26] or GraphPad Prism^®^ software (Prism 5.0) [63,64]) in the appropriate pharmacological preparations.

For changes in spontaneous contractility, all the calculations were performed in a post-processing phase with LabChart software. To avoid errors due to the presence of artifacts, the period of analysis was selected by a skilled operator.

### 4.3. Activity vs. Probiotics

The effectiveness of **62** and **65** against bacteria was evaluated. Compounds **62** and **65** at growing concentrations (0.01, 0.1, 1, and 5 μM) were tested in vitro against lactobacilli (*Lactobacillus rhamnosus* ATCC 7469, IVD Microbiologicals: https://www.microbiologics.com) and bifidobacteria (*Bifidobacterium breve* ATCC 15700, Microbiologicals: https://www.microbiologics.com). The Petri plates used were Rogosa Agar (Liofilchem cod. 10039: https://www.liofilchem.com, acceded on 5 May 2025) for *lactobacilli* and *Bifidobacterium* agar (Liofilchem cod. 10458: https://www.liofilchem.com, acceded on 5 May 2025) for *bifidobacteria*. Incubation times and conditions are detailed in the Instruction for Use (IFU) of the manufacturer. Every test was performed in triplicate, spotting 4 μL, and using DMSO as both the blank and diluent.

## 5. Conclusions

**Figure 8:**
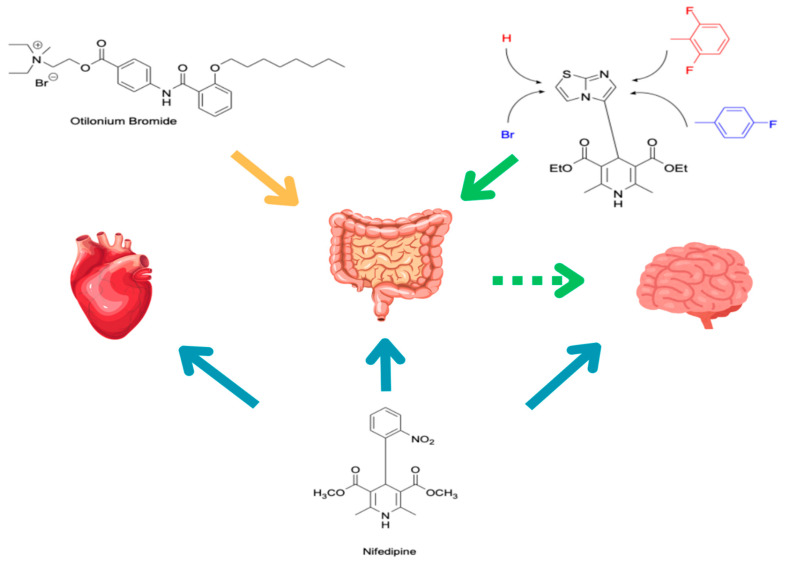
Outlook of conclusion remarks and perspectives.

The present study highlights the potential of selected 1,4-dihydropyridine derivatives, particularly **62** and **65** bearing an imidazo[2,1-*b*]thiazole scaffold, as promising candidates for the treatment of intestinal motility disorders and for irritable bowel syndrome (Figure 8). These compounds demonstrated selective spasmolytic activity on the gastrointestinal smooth muscle, especially the longitudinal layer of the ileum and colon, while lacking significant cardiovascular effects. Their pharmacodynamic profile partially overlaps with that of **OB**, a well-known drug indicated in Roma IV criteria for gut motility disorders.

Moreover, both **62** and **65** showed a favorable impact on spontaneous contractility and did not impair key physiological processes such as propulsion, mixing, or fragmentation at therapeutic concentrations, especially for **62**, both at the ileum and colon levels. Preliminary microbiological assessments further revealed no detrimental effects on key intestinal probiotics such as *Bifidobacterium* and *Lactobacillus* spp., suggesting compatibility with improvements on gut microbiota health, exerting a key role in GBA regulation and inflammatory conditions. In addition, the increase in SCFAs such as butyrate is an essential consideration in GBA regulation and inflammatory conditions. It is known that these bacterial populations increase significantly if the patient follows a diet rich in prebiotic fibers [65], making possible a possible co-administration to amplify the intestinal and systemic effects.

These considerations are supported by recent studies that have demonstrated how the administration of an aqueous probiotic suspension promotes the colonization and growth of probiotic strains in the colon, leading to increased lactate levels, promoting enhanced SCFA production, particularly butyrate [66].

Lastly, previous studies have reported weak pro-apoptotic activity of diethyl 1,4-dihydropyridine derivatives carrying an indole moiety at position 4 in colon cancer cell lines [67], underscoring the importance of further characterizing our newly identified compounds not only in the context of intestinal motility modulation but also for their potential anticancer properties. This multidimensional profile supports the proposed repurposing strategy and warrants further investigation into the mechanisms of action and therapeutic applications of these compounds.

## Figures and Tables

**Table 1:** Relaxant activity of methyl esters compounds on K^+^-depolarized guinea pig ileum longitudinal smooth muscle vs. cardiovascular parameters.

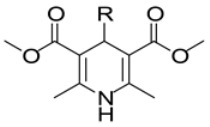							
Compound	Ileum Longitudinal Smooth Muscle				Cardiovascular System *^#^*		
	R	IA *^f^* (M ± SEM)	IC_50_ *^g^* (μM)	95% Conf Lim (μM)	IC_50_ INO *^h^*	IC_50_ CHRONO *^i^*	IC_50_ VR *^j^*
**NIF**		70 ± 0.36 *^k^*	0.0015	0.0011–0.0022	0.26 (0.19–0.36)	0.039 (0.031–0.051)	0.009 (0.003–0.020)
**OB**		90 ± 3.0	8.52	7.14–10.11	0.037 (0.034–0.039)	---	---
**1 *^a^***	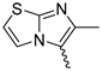	**94 ± 3.2 *^l^***	**1.12**	**0.93–1.36**	**84 ± 2.7 *^s^*** **2.17** **(1.55–3.02)**	**48 ± 1.2 *^t^*** **---**	**30 ± 1.7 *^s^*** **---**
**2 *^b^***	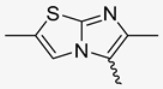	70 ± 1.3	8.83	5.53–10.41	---	---	---
**3 *^b^***	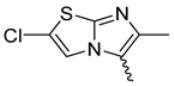	96 ± 3.3 *^l^*	0.55	0.19–0.93	0.13 (0.079–0.20)		
**4 *^b^***	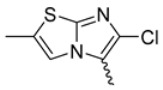	93 ± 0.5	2.06	1.55–2.73	0.056 (0.041–0.076)	---	---
**5 *^b^***	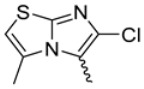	97 ± 0.6 *^l^*	0.51	0.39–0.62	0.10 (0.08–0.15)	---	---
**6 *^b^***	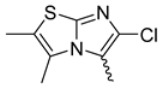	78 ± 1.2	0.32	0.24–0.41	0.081 (0.056–0.11)	---	---
**7 *^b^***	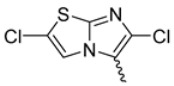	60 ± 1.4 *^m^*	0.68	0.55–0.84	0.18 (0.13–0.23)	---	---
**8 *^a^***	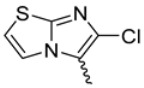	**90 ± 1.4 *^l^***	**1.17**	**0.87–1.88**	**84 ± 2.3 *^t^*** **0.68** **(0.45–1.04)**	**15 ± 0.7 *^t^*** **---**	**15 ± 0.9 *^s^*** **---**
**9 *^b^***	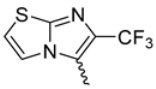	70 ± 2.3 *^n^*	0.21	0.14–0.31	0.071 (0.021–0.14)	0.86 (0.74–1.01)	---
**10 *^b^***	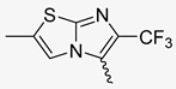	92 ± 1.4 *^m^*	0.095	0.072–0.13	---	---	---
**11 *^c^***	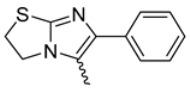	80 ± 1.1 *^m^*	0.26	0.20–0.33	0.39 (0.26–0.61)	8.63 (5.93–10.25)	---
**12 *^b^***	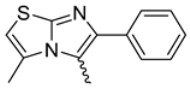	98 ± 1.9	1.04	0.88–1.32	0.83 (0.55–1.21)	1.18 (0.86–1.43)	---
**13 *^b^***	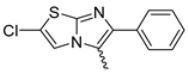	**98 ± 1.6** *^l^*	**0.35**	**0.14–0.83**	1.96 (1.67–2.35)	**4.16** **(3.25–5.34)**	---
**14 *^b^***	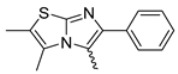	74 ± 3.8	2.96	2.24–3.92	0.59 (0.43–0.81)	**0.66** **(0.51–0.85)**	---
**15 *^b^***	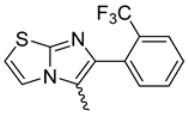	92 ± 1.5	1.66	1.36–2.05	0.093 (0.068–0.12)	---	---
**16 *^b^***	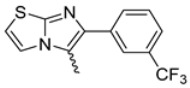	96 ± 1.0 *^l^*	0.036	0.028–0.046	0.31 (0.22–0.42)	**3.76** **(2.92–4.85)**	---
**17 *^c^***	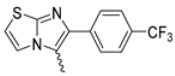	69 ± 3.6 *^o^*	0.0046	0.0036–0.0058	0.12 (0.082–0.17)	---	---
**18 *^a^***	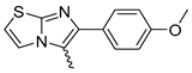	**73 ± 0.8** *^p^*	**0.026**	**0.022–0.031**	**88 ± 3.2** *^s^* **0.59** **(0.41–0.88)**	**84 ± 3.4** *^m^* **0.41** **(0.19–0.79)**	**38 ± 1.4***^l^* ---
**19 *^b^***	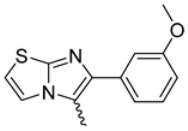	51 ± 2.4 *^q^*	0.083	0.066–0.10	1.90 (1.65–2.27)	1.36 (0.97–1.70)	---
**20 *^b^***	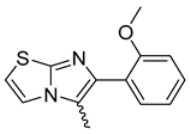	88 ± 2.2 *^l^*	0.86	0.64–1.02	1.97 (1.71–2.31)	4.89 (3.50–6.83)	---
**21 *^c^***	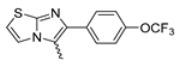	73 ± 3.2 *^o^*	0.0033	0.0025–0.0042	2.33 (1.90–2.66)	---	0.016 (0.012–0.025)
**22 *^b^***	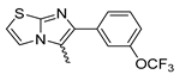	76 ± 1.3 *^p^*	0.018	0.010–0.097	0.83 (0.60–1.04)	**2.41** **(1.85–3.15)**	---
**23 *^b^***	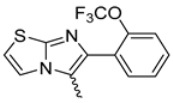	81 ± 3.2 *^l^*	1.95	1.54–2.47	0.075 (0.050–0.093)	---	---
**24 *^b^***	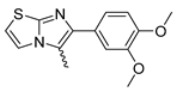	90 ± 2.3 *^m^*	0.45	0.10–0.91	2.64 (2.03–3.01)	**3.01** **(2.41–3.76)**	---
**25 *^d^***	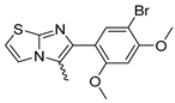	93 ± 2.5 *^l^*	1.18	0.97–1.43	0.30 (0.27–0.39)	1.67 (1.35–1.98)	---
**26 *^b^***	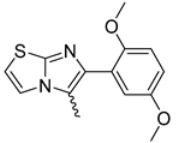	51 ± 0.6	12.35	8.56–17.80	0.44 (0.29–0.65)	---	---
**27 *^b^***	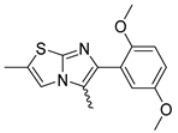	74 ± 1.4	4.89	3.74–6.40	1.43 (1.02–1.94)	**6.62** **(4.37–10.02)**	
**28 *^b^***	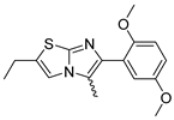	92 ± 1.1	2.16	1.03–2.94	2.24 (1.85–2.43)	**1.59** **(1.26–2.02)**	---
**29 *^b^***	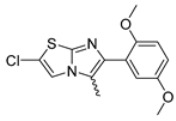	91 ± 2.3 *^n^*	1.14	0.73–1.85	0.90 (0.56–1.44)	0.98 (0.73–1.33)	---
**30 *^b^***	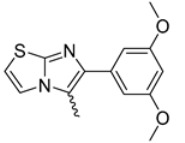	89 ± 3.5 *^r^*	0.055	0.043–0.070	---	**1.38** **(1.08–1.78)**	---
**31 *^b^***	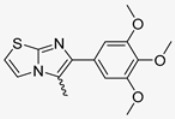	64 ± 2.6 *^m^*	0.55	0.43–0.69	---	---	---
**32 *^b^***	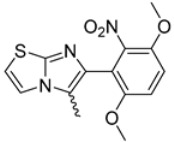	86 ± 5.2 *^s^*	11.04	8.18–14.90	0.36 (0.25–0.51)	---	---
**33 *^b^***	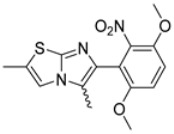	67 ± 3.2 *^l^*	1.84	1.49–2.26	0.093 (0.063–0.14)	---	---
**34 *^b^***	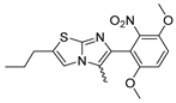	89 ± 2.4 *^s^*	12.81	10.00–16.41	0.039 (0.030–0.051)	---	---
**35 *^b^***	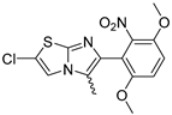	88 ± 1.4	1.83	1.44–2.33	---	18.52 (14.19–22.16)	---
**36 *^b^***	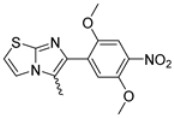	97 ± 1.4	2.56	1.90–3.11	0.054 (0.036–0.079)	6.08 (5.19–7.12)	---
**37 *^b^***	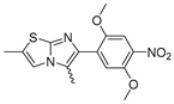	94 ± 3.5 *^m^*	0.24	0.19–0.29	0.026 (0.018–0.036)	3.59 (2.71–4.76)	---
**38 *^b^***	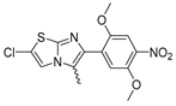	94 ± 1.4 *^s^*	2.56	1.90–3.11	0.36 (0.22–0.59)	**0.69** **(0.51–0.95)**	---
**39 *^b^***	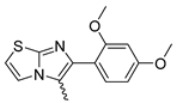	**65 ± 1.2** *^l^*	**2.36**	**1.75–3.19**	1.24 (0.93–1.48)	**8.60** **(7.47–9.90)**	---
**40 *^b^***	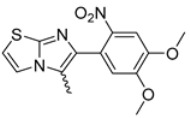	86 ± 1.3 *^n^*	11.43	8.78–14.87	1.34 (0.94–1.88)	**12.37** **(10.00–15.32)**	---
**41 *^e^***	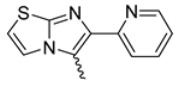	87 ± 2.4	2.57	1.97–3.35	0.54 (0.36–0.79)	24.39 (18.50–29.21)	---
**42 *^e^***	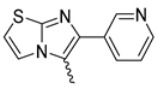	91 ± 0.4	3.55	2.05–6.16	---	28.44 (21.38–36.96)	---
**43 *^e^***	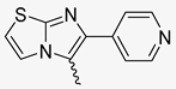	90 ± 2.1 *^l^*	0.96	0.74–1.06	---	---	---
**44 *^e^***	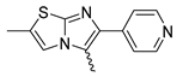	97 ± 0.5 *^s^*	9.69	7.61–12.35	---	7.49 (3.52–9.91)	---
**45 *^e^***	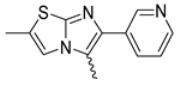	83 ± 2.5 *^n^*	15.96	10.93–20.10	---	12.46 (9-67–16.10)	---
**46 *^e^***	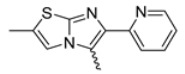	78 ± 2.1	2.81	2.20–3.57	---	39.91 (32.71–45.72)	---
**47 *^e^***	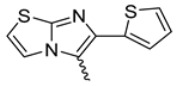	91 ± 0.3 *^r^*	0.11	0.086–0.13	---	1.30 (1.04–1.62)	---
**48 *^e^***	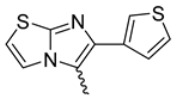	85 ± 1.7 *^q^*	0.0023	0.0018–0.0030	---	1.06 (0.80–1.41)	---
**49 *^e^***	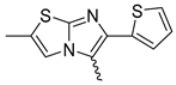	81 ± 2.3 *^l^*	1.37	1.08–1.72	---	40.50 (31.62–48.87)	---
**50 *^e^***	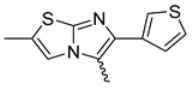	74 ± 2.6	3.52	2.68–4.61	---	2.30 (1.74–3.04)	---
**51 *^e^***	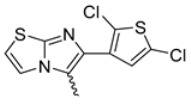	95 ± 2.6 *^r^*	0.18	0.10–0.31	0.046 (0.032–0.064)	2.16 (1.83–2.55)	---
**52 *^e^***	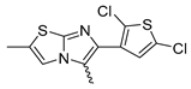	87 ± 1.1	3.23	2.39–4.36	0.31 (0.093–0.81)	---	---

**NIF** (Nifedipine). **OB** (Otilonium Bromide). *^a^* First published in [24]. *^b^* First published in [17]. *^c^* First published in [18]. *^d^* First published in [19]. *^e^* First published in [20]. The data are taken from references reported as indicated. **Bold**: unpublished data. **Background color**: compounds with no noteworthy cardiovascular activity. ^#^ For compounds **1**, **8**, and **18**, data on these parameters have never been published; IA is also reported. *^f^* **IA** (Intrinsic Activity) expressed as percent inhibition of calcium-induced contraction on K^+^-depolarized (80 mM) guinea pig ileum longitudinal smooth muscle at 10 μM. The 10 μM concentration gave the maximum effect for most compounds. *^g^* Calculated from log concentration–response curves (Probit analysis by Litchfield and Wilcoxon [26] with *n* = 6–7). *^h^* **IC_50_ INO** (Potency of negative inotropy calculated as decrease in developed tension on isolated guinea pig left atrium driven at 1 Hz).; *^i^* **IC_50_ CHRONO** (Potency of negative chronotropy calculated on guinea pig spontaneously beating isolated right atrium, expressed as percent in atrial rate changes from the control. Pretreatment heart rate ranged from 165 to 190 beats/min); *^j^* **IC_50_ VR** [Potency of vasorelaxant effect calculated as inhibition of calcium-induced contraction on K^+^-depolarized (80 mM) guinea pig aortic strips]. When the maximum IA for cardiovascular parameters was <50% (**---**), the IC_50_ was not reported. *^#^* For compounds **1**, **8**, and **18**, complete cardiovascular data have been included as they have never been published. *^k^* At 0.005 μM. *^l^* At 5 μM. *^m^* At 1 μM. *^n^* At 50 μM. *^o^* At 0.01 μM. *^p^* At 0.05 μM. *^q^* At 0.1 μM. *^r^* At 0.5 μM. *^s^* At 100 μM. *^t^* At 10 μM.

**Table 2:** Relaxant activity of ethyl esters compounds on K^+^-depolarized guinea pig ileum longitudinal smooth muscle vs. cardiovascular parameters.

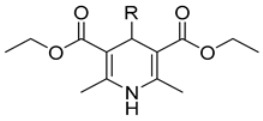							
Compound	Ileum Longitudinal Smooth Muscle				Cardiovascular System		
	R	IA *^d^* (M ± SEM)	IC_50_ *^e^* (μM)	95% Conf Lim (μM)	IC_50_ INO *^f^*	IC_50_ CHRONO *^g^*	IC_50_ VR *^h^*
**NIF**		70 ± 0.36 *^i^*	0.0015	0.0011–0.0022	0.26 (0.19–0.36)	0.039 (0.031–0.051)	0.009 (0.003–0.020)
**OB**		90 ± 3.0	8.52	7.14–10.11	0.037 (0.034–0.039)	---	---
**53 *^a^***	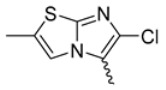	94 ± 1.4 *^j^*	0.057	0.043–0.076	0.031 (0.024–0.039)	---	---
**54 *^a^***	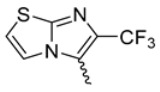	90 ± 1.4 *^k^*	0.014	0.010–0.018	1.07 (0.76–1.46)	---	---
**55 *^a^***	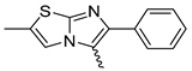	83 ± 2.4 *^l^*	0.25	0.19–0.32	---	7.15 (4.30–11.88)	---
**56 *^a^***	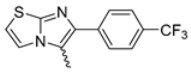	83 ± 1.3 *^k^*	0.00088	0.00021–0.0017	0.033 (0.026–0.044)	0.15 (0.071–0.67)	---
**57 *^a^***	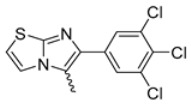	77 ± 0.2 *^l^*	0.038	0.029–0.050	0.43 (0.31–0.57)	0.61 (0.41–0.89)	---
**58 *^b^***	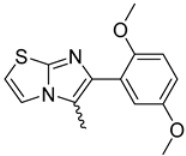	85 ± 2.3 *^m^*	0.046	0.029–0.071	1.16 (0.85–1.68)		---
**59 *^c^***	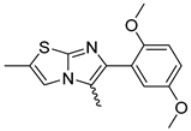	**92 ± 2.2**	**1.17**	**0.91–1.50**	0.25 (0.18–0.36)	4.81 (4.01–5.03)	---
**60 *^c^***	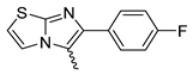	**93 ± 0.5** *^k^*	**0.015**	**0.011–0.019**	0.064 (0.046–0.088)	1.65 (1.36–2.00)	---
**61 *^c^***	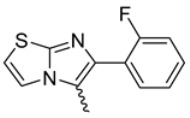	**99 ± 0.1**	**0.47**	**0.36–0.64**	5.15 (3.27–8.09)	0.98 (0.53–1.80)	---
**62 *^c^***	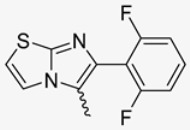	**92 ± 1.4**	**1.92**	**1.54–2.39**	---	---	---
**63 *^c^***	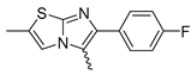	**90 ± 0.8**	**1.63**	**1.12–2.19**	---	0.023 (0.0091–0.043)	---
**64 *^c^***	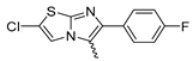	**94 ± 2.2** *^j^*	**0.36**	**0.26–0.51**	0.049 (0.031–0.077)	1.73 (1.31–2.30)	---
**65 *^c^***	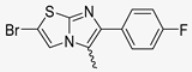	**97 ± 0.9** *^j^*	**0.62**	**0.48–0.77**	---	---	---
**66 *^c^***	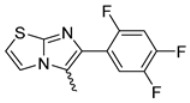	**94 ± 2.6** *^j^*	**0.32**	**0.24–0.42**	0.16 (0.11–0.23)	1.24 (1.06–1.45)	5.57 (3.94–7.83)
**67 *^c^***	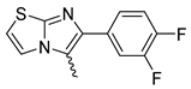	**97 ± 1.1** *^m^*	**0.018**	**0.013–0.024**	0.021 (0.016–0.027)	0.10 (0.085–0.12)	---
**68 *^c^***	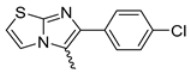	**97 ± 3.3** *^n^*	**0.0023**	**0.0017–0.0029**	0.097 (0.048–0.19)	---	---
**69 *^c^***	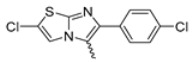	**82 ± 3.2** *^l^*	**0.21**	**0.16–0.26**	3.50 (2.70–4.52)	1.73 (1.17–2.54)	---
**70 *^c^***	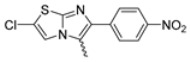	**90 ± 3.5** *^p^*	**0.44**	**0.31–0.63**	1.37 (0.98–1.93)	---	---
**71 *^c^***	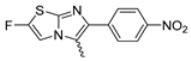	**90 ± 1.7** *^m^*	**0.058**	**0.042–0.082**	0.024 (0.018–0.030)	---	---
**72 *^c^***	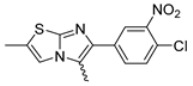	**98 ± 1.0** *^j^*	**0.35**	**0.27–0.46**	0.19 (0.14–0.26)	8.48 (6.73–10.04)	---
**73 *^c^***	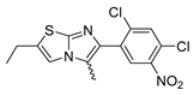	**99 ± 0.1**	**0.33**	**0.26–0.40**	0.033 (0.025–0.041)	---	---
**74 *^c^***	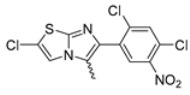	**97 ± 2.3** *^o^*	**0.28**	**0.19–0.41**	0.081 (0.056–0.11)	---	---
**75 *^c^***	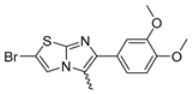	**93 ± 1.5** *^j^*	**0.45**	**0.32–0.65**	---	0.31 (0.25–0.38)	---
**76 *^c^***	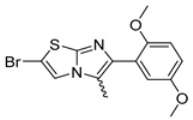	**85 ± 3.7** *^l^*	**0.31**	**0.25–0.39**	0.026 (0.017–0.041)	0.12 (0.097–0.15)	---
**77 *^c^***	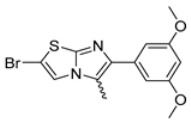	**89 ± 3.2**	**1.00**	**0.77–1.30**	0.025 (0.019–0.031)	0.64 (0.50–0.81)	---
**78 *^c^***	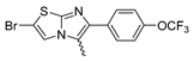	**87 ± 2.4** *^j^*	**1.00**	**0.85–1.17**	0.093 (0.062–0.13)	---	---
**79 *^c^***	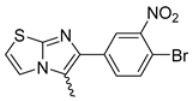	**98 ± 1.2** *^p^*	**0.0011**	**0.00083–0.0025**	1.27 (0.92–1.76)	0.0043 (0.0039–0.0067)	---

**NIF** (Nifedipine). **OB** (Otilonium Bromide). *^a^* First published in [18]. *^b^* First published in [20]. *^c^* First published in [19]. The data are taken from references reported as indicated. **Bold**: unpublished data. **Background color**: compounds with no notable cardiovascular activity. *^d^ ***IA** (Intrinsic Activity) expressed as percent inhibition of calcium-induced contraction on K^+^-depolarized (80 mM) guinea pig ileum longitudinal smooth muscle at 10 μM. The 10 μM concentration gave the maximum effect for most compounds. *^e^* Calculated from log concentration–response curves (Probit analysis by Litchfield and Wilcoxon [26] with *n* = 6–7). *^f^* **IC_50_ INO** (Potency of negative inotropy calculated as decrease in developed tension on isolated guinea pig left atrium driven at 1 Hz).; *^g^* **IC_50_ CHRONO** (Potency of negative chronotropy calculated on guinea pig spontaneously beating isolated right atrium, expressed as percent in atrial rate changes from the control. Pretreatment heart rate ranged from 165 to 190 beats/min); *^h^* **IC_50_ VR** [Potency of vasorelaxant effect calculated as inhibition of calcium-induced contraction on K^+^-depolarized (80 mM) guinea pig aortic strips]. When the maximum IA for cardiovascular parameters was <50% (**---**), the IC_50_ was not reported. *^i^* At 0.005 μM. *^j^* At 5 μM. *^k^* At 0.1 μM. *^l^* At 1 μM. *^m^* At 0.5 μM. *^n^* At 0.05 μM. *^o^* At 50 μM. *^p^* At 0.01 μM.

**Table 3:** Relaxant activity of allyl esters compounds on K^+^-depolarized guinea pig ileum longitudinal smooth muscle vs. cardiovascular parameters.

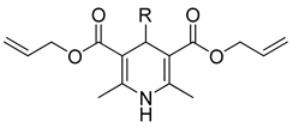							
Compound	Ileum Longitudinal Smooth Muscle				Cardiovascular System		
	R	IA *^c^* (M ± SEM)	IC_50_ *^d^* (μM)	95% Conf Lim (μM)	IC_50_ INO *^e^*	IC_50_ CHRONO *^f^*	IC_50_ VR *^g^*
**NIF**		70 ± 0.36 *^h^*	0.0015	0.0011–0.0022	0.26 (0.19–0.36)	0.039 (0.031–0.051)	0.009 (0.003–0.020)
**OB**		90 ± 3.0	8.52	7.14–10.11	0.037 (0.034–0.039)	---	---
**80 *^a^***	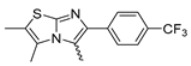	72 ± 2.3 *^i^*	0.46	0.36–0.61	0.27 (0.21–0.36)	8.96 (7.65–10.50)	---
**81 *^a^***	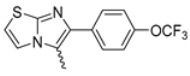	90 ± 3.4 *^j^*	0.0053	0.0038–0.0073	0.73 (0.51–1.01)	1.97 (1.01–3.54)	---
**82 *^a^***	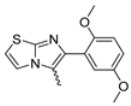	96 ± 2.5 *^k^*	0.32	0.24–0.42	0.34 (0.23–0.48)	---	---
**83 *^b^***	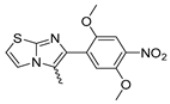	**95 ± 0.7**	**1.08**	**0.84–1.41**	0.025 (0.019–0.032)	2.38 (1.92–2.83)	---

**NIF** (Nifedipine). **OB** (Otilonium Bromide). *^a^* First published in [18]. *^b^* First published in [19]. The data are taken from references reported as indicated. **Bold**: unpublished data. *^c^* **IA** (Intrinsic Activity) expressed as percent inhibition of calcium-induced contraction on K^+^-depolarized (80 mM) guinea pig ileum longitudinal smooth muscle at 10 μM. The 10 μM concentration gave the maximum effect for most compounds. *^d^* Calculated from log concentration–response curves (Probit analysis by Litchfield and Wilcoxon [26] with *n* = 6–7). *^e^* **IC_50_ INO** (Potency of negative inotropy calculated as decrease in developed tension on isolated guinea pig left atrium driven at 1 Hz.); *^f^* **IC_50_ CHRONO** (Potency of negative chronotropy calculated on guinea pig spontaneously beating isolated right atrium, expressed as percent in atrial rate changes from the control. Pretreatment heart rate ranged from 165 to 190 beats/min); *^g^* **IC_50_ VR** [Potency of vasorelaxant effect calculated as inhibition of calcium-induced contraction on K^+^-depolarized (80 mM) guinea pig aortic strips]. When the maximum IA for cardiovascular parameters was <50% (**---**), the IC_50_ was not reported. *^h^* At 0.001 μM. *^i^* At 1 μM. *^j^* At 0.05 μM. *^k^* At 5 μM.

**Table 4:** Spasmolytic activity of selected compounds on K^+^-depolarized guinea pig longitudinal colon smooth muscle.

Compound	IA *^a^* (M ± SEM)	IC_50_ *^b^* (μM)	95% Conf Lim (μM)
**NIF**	94 ± 1.6 *^c^*	0.0019	0.0015–0.0024
**OB**	90 ± 2.3 *^d^*	3.43	2.65–4.44
**2**	**97 ± 2.4**	**0.20**	**0.15–0.26**
**10**	**89 ± 1.7**	**2.46**	**1.94–3.14**
**31**	**64 ± 2.3 *^e^***	**0.30**	**0.23–0.37**
**43**	**82 ± 2.4 *^e^***	**0.10**	**0.08–0.13**
**62**	**78 ± 2.4 *^f^***	**1.75**	**1.39–2.20**
**65**	**96 ± 1.3 *^g^***	**0.049**	**0.035–0.068**

**NIF** (Nifedipine). **OB** (Otilonium Bromide). *^a^* **IA** (Intrinsic Activity) expressed as percent inhibition of calcium-induced contraction on K^+^-depolarized (80 mM) guinea pig colon longitudinal smooth muscle at 10 μM. **Bold**: unpublished data. The 10 μM concentration gave the maximum effect for most compounds. *^b^* Calculated from log concentration–response curves (Probit analysis by Litchfield and Wilcoxon [26] with *n* = 6–7). When the maximum IA was <50%, the IC_50_ was not calculated. *^c^* At 0.01 μM. *^d^* At 50 μM. *^e^* At 0.5 μM. *^f^* At 5 μM. *^g^* At 1 μM.

**Table 5:** Spasmolytic activity of selected compounds on K^+^-depolarized guinea pig circular ileum and colon smooth muscle.

Compound	IA *^a^* (M ± SEM)	IC_50_ *^b^* (μM)	95% Conf Lim (μM)	IA *^a^* (M ± SEM)	IC_50_ *^b^* (μM)	95% Conf Lim (μM)
**NIF**	84 ± 1.3 *^c^*	0.00028	0.00020–0.00041	60 ± 2.4 *^d^*	0.11	0.079–0.16
**OB**	3 ± 0.2 *^d^*			10 ± 0.3 *^d^*		
**2**	**98 ± 2.1 *^e^***	**0.067**	**0.018–0.025**	**33 ± 1.6 *^e^***		
**10**	**72 ± 1.6**	**2.64**	**2.09–3.32**	**59 ± 2.2**	**1.68**	**1.15–2.47**
**31**	**78 ± 2.1 *^f^***	**2.68**	**2.10–3.22**	**67 ± 1.4**	**1.20**	**0.89–1.40**
**43**	**83 ± 2.2 *^g^***	**0.013**	**0.010–0.017**	**63 ± 1.8**	**0.50**	**0.35–0.77**
**62**	**15 ± 0.3 *^f^***			**44 ± 1.9**		
**65**	**2 ± 0.1**			**41 ± 1.6**		

**NIF** (Nifedipine). **OB** (Otilonium Bromide). **Bold**: unpublished data. **Background color**: compounds with activity in line with that of the functional analogue **OB**. *^a^* **IA** (Intrinsic Activity) expressed as percent inhibition of calcium-induced contraction on K^+^-depolarized (80 mM) guinea pig ileum and colon circular smooth muscle at 50 μM. The 50 μM concentration gave the maximum effect for most compounds. *^b^* Calculated from log concentration–response curves (Probit analysis by Litchfield and Wilcoxon [26] with *n* = 6–7). When the maximum effect was <50%, the IC_50_ values were not calculated.*^c^* At 0.01 μM. *^d^* At 0.05 μM. *^e^* At 0.1 μM. *^f^* At 5 μM. *^g^* At 0.05 μM.*^.^*

**Table 6:** Effects on the ileum of the different compounds on transit speed, pain, mixing, fragmentation, and tones.

Compound	Conc. (µM)	Transit Speed Variation %	Pain %	Mixing %	Fragmentation %	Longitudinal Contraction Variation %	Circular Contraction Variation %
**NIF**	0.01	=	=	−	=	−	+
	0.05	=	=	−	=	−	−
	0.1	=	=	−	−	−	−
	0.5	=	=	−	=	−	−
	1	−	=	−	−	−	−
	5	−	−	−	−	−	−
	10	−	−	−	=	−	−
**OB**	0.01	=	=	+	+	− −	+
	0.05	=	=	=	+	− −	−
	0.1	=	−	=	+	−–−	−
	0.5	=	−	=	+	−–−	−
	1	=	−	+	+	−–−	−
	5	−	−	+	+	−–− −	−
	10	−	−	+	−	−–− −	−
**62**	0.01	=	=	=	=	=	+
	0.05	=	=	=	=	+	−
	0.1	=	=	=	=	+	−
	0.5	=	=	=	=	−	−
	1	−	−	=	−	−–−	−
	5	−	−	=	−	−–−	−
	10	−	−	−	−	−–−	−
**65**	0.01	=	=	=	=	=	+
	0.05	=	=	−	=	=	−
	0.1	=	=	=	=	=	−
	0.5	=	=	=	=	−	−
	1	−	−	=	=	−	−
	5	−	=	=	=	−	−
	10	−	−	=	=	−	−

**Transit speed variation** %, **Pain** %, **Mixing** %, **Fragmentation** %: ≤–500%: “−–−”; ]–500%,–300%]: “− −”; ]–300%,–50%]: “−”; ]–50%,+50%[: “=”; [+50%,+300%[: “+”; [+300%,+500%[: “++”; ≥+500%: “+++”. **Longitudinal and circular contraction**: ≤–100%: “−–−–− −”; ]–100%,–80%]: “−–−–−”; ]–80%,–60%]: “−–− −”; ]–60%,–40%]: “−–−”; ]–40%,–20%]: “− −”; ]–20%,0%[: “−”; NS (Not Significant): “=”; ]0%,+20%[: “+”; [+20%,+40%[: “++”; [+40%,+60%[: “+++”; [+60%,+80%[: “++++”; [+80%,+100%[: “+++++”; ≥+100%: “++++++”.

**Table 7:** Effects on the colon of the different compounds on transit speed, pain, mixing, fragmentation, and tones.

Compound	Conc. (µM)	Transit Speed Variation %	Pain %	Mixing %	Fragmentation %	Longitudinal Contraction Variation %	Circular Contraction Variation %
**NIF**	0.01	=	=	=	=	−	−
	0.05	=	=	−	=	−	−
	0.1	=	=	−	−	−	−
	0.5	=	=	−	=	−	−
	1	=	=	=	=	−	−
	5	=	=	−	−	−	−
	10	=	=	/	/	/	/
**OB**	0.01	=	=	=	+	=	=
	0.05	=	=	=	=	=	=
	0.1	=	=	=	=	=	=
	0.5	=	=	=	=	=	=
	1	=	=	=	=	=	=
	5	=	=	=	=	−	=
	10	=	=	−	−	−	=
**62**	0.01	++	+	+	+	−	+
	0.05	=	=	+	+	− −	+
	0.1	=	++	=	=	− −	+
	0.5	=	+	−	=	− −	+
	1	=	+	=	=	−–−	+
	5	+	+	−	−	−–−	+
	10	++	+	−	−	−–− −	+
**65**	0.01	=	=	=	=	−	−
	0.05	=	=	=	−	−	−
	0.1	=	=	=	−	−	−
	0.5	−	=	−	−	− −	−
	1	−	=	−	−	− −	−
	5	−	=	−	−	−–−	−
	10	−	=	−	=	−–−	−

**Transit speed variation** %, **Pain** %, **Mixing** %, **Fragmentation** %: ≤–500%: “−–−”; ]–500%,–300%]: “− −”; ]–300%,–50%]: “−”; ]–50%,+50%[: “=”; [+50%,+300%[: “+”; [+300%,+500%[: “++”; ≥+500%: “+++”. **Longitudinal and circular contraction**: ≤–100%: “−–−–− −”; ]–100%,–80%]: “−–−–−”; ]–80%,–60%]: “−–− −”; ]–60%,–40%]: “−–−”; ]–40%,–20%]: “− −”; ]–20%,0%[: “−”; Not Significant: “=”; ]0%,+20%[: “+”; [+20%,+40%[: “++”; [+40%,+60%[: “+++”; [+60%,+80%[: “++++”; [+80%,+100%[: “+++++”; ≥+100%: “++++++”. /: not applicable.

## Data Availability

Data presented in this study is contained within the article. Further inquiries can be directed to the corresponding author.

## Abbreviations

The following abbreviations are used in this manuscript:

BSMA	Basal Spontaneous Motor Activity
CNS	Central Nervous System
DHP	Dihydropyridine
FFT	Fast Fourier Transform
GBA	Gut–Brain Axis
HF	High-Frequency
IC_50_	Half maximal inhibitory concentration
LF	Low Frequency
MCA	Mean Contraction Amplitude
MF	Medium Frequency
OB	Otilonium Bromide
PSS	Physiological Salt Solution
PSD	Power Spectral Density
SC	Spontaneous Contraction
SCV	Spontaneous Contraction Variability
VDCC	Voltage-Dependent Calcium Channel
